# Repeatability of the 6-min walk test in non-cystic fibrosis bronchiectasis

**DOI:** 10.1038/s41598-020-75093-7

**Published:** 2020-11-05

**Authors:** Paula Maria Eidt Rovedder, Rafael Oliveira Fernandes, Patrícia Santos Jacques, Bruna Ziegler, Francini Porcher Andrade, Paulo de Tarso Roth Dalcin

**Affiliations:** 1 Programa de Pós-Graduação em Ciências Pneumológicas, Universidade Federal do Rio Grande do Sul (UFRGS), Porto Alegre, Brazil; 2 Escola de Educação Física, Fisioterapia e Dança (ESEFID), UFRGS, Porto Alegre, Brazil; 3 Programa de Pós-Graduação em Saúde da Criança e do Adolescente (PPGSCA), UFRGS, Rua Ramiro Barcelos, 2400, sala 220, Porto Alegre, 90035-003 Brazil; 4 Serviço de Fisioterapia, Hospital de Clínicas de Porto Alegre (HCPA), Porto Alegre, Brazil; 5 Serviço de Pneumologia, HCPA, Porto Alegre, Brazil

**Keywords:** Medical research, Respiratory tract diseases, Diagnosis, Rehabilitation

## Abstract

Non-cystic fibrosis bronchiectasis (NCFB) is a chronic lung disease characterized by progressive and irreversible changes of the bronchial tree. The evaluation of exercise capacity is essential to manage this disease. This study aims to determine the within-subject repeatability of two Six Minute Walk Test (6MWT) in adults with NCFB. NCFB. This cross-sectional observational study included 66 NCFB subjects above 18 years-old (mean of 55 ± 17 years old, 68% women). 73% of the participants presented moderate to severe clinical condition classified by Bronchiectasis Severity Index. It showed that these participants walked 16.6 m less (95%CI 3.8 to 29.4; p < 0.01) in the second 6MWT when compared to the first test, with a within-subject coefficient variation of 9.4% (95%CI 7.2–11.2%) and an intra-test reliability with a high intraclass correlation coefficient of 0.88 (95%CI 0.80–0.93). Bland–Altman plot showed an agreement regarding test repeatability, besides presented a large limit of agreement (− 85 to 116 m). Respiratory rate and systolic blood pressure were significantly higher before starting the second test. In conclusion, 6MWT seems to be reproducible in NCFB subjects and vital sign verification should be attentively checked to assess if the patient is fully recovered to perform a second test, as well as the disease severity score. Other studies on this matter should be conducted with a larger number of participants to confirm the findings of the present study.

## Introduction

Non-cystic fibrosis bronchiectasis (NCFB) is a chronic lung disease characterized by progressive and irreversible changes of the bronchial tree due to persistent inflammatory processes in the airways. Its pathophysiology is based on the permanent and abnormal dilation of the bronchi and bronchioles^1^.

The main clinical manifestation of bronchiectasis is a chronic productive cough^2^. The disease can also be associated with nonspecific symptoms, including general malaise, fatigue, weight loss, dyspnea, and not uncommonly, hemoptysis^2^. Subjects with NCFB commonly experience progressive exercise limitation and a reduction in their activities of daily living^3^. The measurement of exercise capacity is part of the assessment and monitoring of the study subjects and may also provide prognostic information and ease up treatment decisions, including timing of referral for a lung transplantation^4^.

The Six Minute Walk Test (6MWT) is an easy, well tolerated and inexpensive method to evaluate cardiopulmonary functional capacity and it is widely used in clinical practice in order to evaluate exercise limitation in chronic pulmonary disease^5^. The 6MWT reflects the ability of individuals to perform activities of daily living^5,6^. The assessment of submaximal tolerance in 6MWT is an important prognostic factor in chronic respiratory diseases^5^.

European Respiratory Society (ERS) and American Thoracic Society (ATS) recommend to perform test–retest with an one-hour interval to assess its repeatability to minimize intra-day variability and maximize learning process^5^. The benefit of performing the second test is the improved coordination by finding the optimal stride length and overcoming anxiety^5^.

Despite the ATS statement about 6MWT, there is a shortage of data in the literature on the repeatability of 6MWT in NCFB patients. The lack of awareness about the repeatability, makes it difficult to determine, in individual cases, if an apparent change could result from the fact that an individual has become more familiar with the procedure and thereby more comfortable while doing the second test. Hence additional studies in NCFB are needed to quantify the magnitude and extent of this variation along with repeated measurements. The primary objective of this study was to determine the within-subject repeatability of the 6MWT in adult subjects with NCFB (2 tests performed following a rest period of 60 min).

## Methods

This was a cross-sectional observational study with subjects diagnosed with NCFB. The results are part of the database from a previous study of the group^7^. The data collection was done at Hospital de Clínicas de Porto Alegre (HCPA—a large, tertiary care, university-affiliated hospital in Southern Brazil) over a 24-month period. The study was approved by Research Ethics Committee at the Hospital de Clínicas de Porto Alegre (process number #08096). All research was performed in accordance with relevant guidelines and regulations. All participants read and signed the Free Informed Consent Term.

Adults above 18 years-old diagnosed with NCFB based on clinical and image criteria (X-ray and CT scan)^1^ where invited to participate. Inclusion criteria: subjects with at least one chronic or recurrent respiratory clinical symptom, such as cough, sputum production, dyspnea, hemoptysis, recurrent respiratory infection, for at least 1 year, pulmonary function with Forced Expiratory Volume 1 Second (FEV_1_) ≤ 70% of the predicted value, clinical stability defined as not having exacerbations or changes in the clinical condition in the last 30 days, and no requirements of hospital admission or changes in treatment regimen. Exclusion criteria: subjects with cystic fibrosis (confirmed or presumed diagnosis), pregnancy or those who were unable to perform the 6MWT.

Bronchiectasis Severity Index (BSI) was used to assess the severity of clinical condition of NCFB patients. This score was calculated based on the sum of 9 variables (age, body mass index, FEV1% predicted, hospitalization or exacerbation before study, degree of dyspnea, chronic colonization by *pseudomonas aeruginosa* or other microorganism, and radiological extension of the disease). According to the overall score, that range from 0 to 26 points, patients were classified into low BSI (0–4 points), intermediate BSI (5–8 points) and high BSI (≥ 9 points)^8^.

The 6MWTs were performed according to the ATS guideline^5,9^. In a 30-m corridor, the subjects were instructed to walk as far as possible for 6 min. The measurements included: walk distance, oxygen saturation (SpO_2_, finger probe), heart rate, respiratory rate, manual blood pressure, and BORG scale (dyspnea and leg fatigue), before and immediately after test. The second 6MWT was performed in the same manner as the first, following a rest period of 60 min. It was recommended that subjects did not exercise vigorously within the 2 h before the test. Since for many patients with severe disease the 6MWT could constitute a vigorous exercise, the ATS/ERS recommendations were carefully followed concerning the rest and interval periods in order to perform both 6MWTs in the same day and place^5,9^. The predicted values for the 6MWT were calculated based on the equation described by Enright and Sherrill^10^. All the 6MWTs were conducted by the same investigator and the subjects did not have previously performed a walk test at the Pneumology Ambulatory Care.

Spirometry was performed with Master Screen (Jaeger V4.31, Würzburg, Germany), following the guideline of the Sociedade Brasileira de Pneumologia e Tisiologia (SBPT)^11^. All tests were performed in the Serviço de Pneumologia at HCPA. Participants performed three spirometric maneuvers, and the ventilatory maneuver that presented the highest values of FEV_1_ and Forced Vital Capacity (FVC) were considered for the study. The spirometric test was performed in the same week of the 6MWT. Results were presented in liters and percentage of predicted value for age, sex, and height.

### Statistical analyses

Statistical analyses were performed using SPSS 22.0. Parametric data were presented as Mean ± Standard Deviation (SD) and non-parametric data as median (interquartile range). Paired t-test or Wilcoxon-signed rank test was used to compare intra-group variation on walked distance and vital signs. Subgroups as age and sex were analyzed by t-test or Mann–Whitney U test. Pearson’s (continuous data) or Spearman’s correlation (continuous or ordinal data) were used to investigate association between variables. Qui-square test was used to compare categorical variable.

The variation between 6MWTs was quantified using the coefficient of variation (CV) (numeric data) and *kappa* coefficient (categorical data) The CV was calculated using the root mean square method, as proposed by Bland^12^. We found the CV for each subject separately, squared them, found their mean, and took the square root of that mean. Confidence intervals were calculated for CV and intraclass correlation coefficient (ICC).

The Bland–Altman method was used to evaluate the difference between the 6MWT distances in relation to the mean of those values individually. The limits of agreement were defined as the average difference of ± 1.96SD. Statistical difference was considered when *p* < 0.05.

## Results

During the period of the study, 70 subjects with a diagnosis of NCFB underwent clinical and functional evaluation. Four subjects were excluded due to interruption of the 6MWT in response to clinical symptoms, such as dyspnea (2 subjects), dizziness (1 subject), and leg fatigue (1 subject). Therefore, 66 subjects were considered for the statistical analysis.

Table 1 presents the characteristics of the subjects. Forty-five were female and 21 were male with a mean age of 54.8 ± 17.5 years old and body mass index 26 ± 5 kg/m^2^. Spirometric values showed an important reduction in FEV_1_ with a median of 1.15 L (range 0.44 to 4.14 L). Tiffeneau-Pinelli Index were also reduced presenting an average of 65.2 ± 16.8. The response to bronchodilator, considered when FEV_1_ increases > 12%, was observed in 35% of the subjects. The severity of disease assessed by BSI showed that 27% presented low score, 29% presented intermediate score and 44% presented high score.

**Table 1:** Baseline characteristics of adolescent and adult patients with bronchiectasis.

Subjects	Values	Min–max
n	66	
Sex (men/women)	21/45	
Age, years	54.8 ± 17.5	18–81
Weight, kg	64.9 ± 15.7	39–100
Height, m	1.58 ± 0.09	1.39–1.82
Body Mass Index, kg/m^2^	25.8 ± 5.3	16.4–37.7
**Spirometry**		
FEV_1_, L	1.2 (0.9–1.6)	0.4–4.1
FEV_1_, % predict	46.1 ± 13.9	14.4–68.8
FVC, L	1.90 (1.5–2.5)	0.8–4.9
FVC, % predict	60.5 ± 14.9	28.3–94.3
FEV_1_/FVC index	65.2 ± 16.8	32.6–100
FEV_1_/FVC index, % predict	76.6 ± 18.9	43.4–118.0
**Spirometry post-bronchodilator (BD)**		
FEV_1_ BD, L	1.2 (1.0–1.6)	0.5–3.5
FEV_1_ BD, % predict	50.6 ± 15.4	17.4–84.7
FVC BD, L	2.06 ± 0.70	0.81–4.60
FVC BD, % predict	63.9 ± 15.7	31.9–108.8
**BSI**		
Low score	18 (27%)	
Intermediate score	19 (29%)	
High score	29 (44%)	

Data presented as mean ± SD, Median (25–75) or stated otherwise.

FEV_1_: forced expiratory volume 1sec; FVC: forced vital capacity; BSI: Bronchiectasis Severity Index.

Table 2 shows a reduction in walked distance in the second test when compared to the first test of 16.6 m (95%CI 3.8 to 29.4) (*p* = 0.011). Despite the significant reduction in distance, the within-subject CV between tests was 9.4% (95%CI 7.2 to 11.2) and presented a high intra-test reliability with an ICC of 0.88 (95%CI 0.80 to 0.93). SpO_2_ and RR were higher in the subjects during the rest period before starting the second 6MWT when compared to the rest period of the first test (*p* = 0.037 and *p* = 0.034, respectively), as well as systolic BP (*p* = 0.000). Significant increase in blood pressure was also observed at the end of second test (*p* = 0.05).

**Table 2:** 6-min-walk test results and reproducibility.

Variable	First 6MWT	Second 6MWT	CV (95%CI), %	ICC (95%CI)
Distance, m	434 ± 78	417 ± 88	9.4 (7.2 to 11.2)	0.88 (0.80 to 0.93)
HR at-rest, beats/min	87 ± 14	88 ± 13	5.7 (3.1 to 7.4)	0.92 (0.88 to 0.95)
HR post-test, beats/min	112 ± 19	113 ± 19	10.2 (4.5 to 13.7)	0.80 (0.68 to 0.88)
RR at-rest, breath/min	19 ± 4	20 ± 3^†^	8.8 (6.8 to 10.4)	0.90 (0.83 to 0.94)
RR post-test, breath/min	25 ± 5	26 ± 5	8.2 (6.2 to 9.8)	0.90 (0.83 to 0.94)
SpO_2_ at-rest, %	95 (94–97)	96 (95–98)^†^	1.0 (0.8 to 1.2)	0.87 (0.79 to 0.95)
SpO_2_ post-test, %	95 (92–97)	95 (92–97)	2.6 (1.3 to 3.4)	0.85 (0.76 to 0.91)
Level O_2_ desaturation, %	− 1 (− 3 to 0)	− 1 (− 3 to 0)	133 (97.5 to 161)	0.75 (0.58 to 0.84)
Desaturation, n (%)^††^	12 (18%)	13 (20%)		
SBP at rest, mmHg	123 ± 17	132 ± 24^†^	7.1 (3.2 to 9.5)	0.80 (0.59 to 0.90)
DBP at rest, mmHg	75 ± 15	75 ± 16	10.2 (3.4 to 4.1)	0.89 (0.83 to 0.94)
SBP post-test, mmHg	125 ± 20	131 ± 24^†^	6.8 (4.5 to 8.4)	0.88 (0.75 to 0.94)
DBP post-test, mmHg	75 ± 15	78 ± 12^†^	16.8 (11.0 to 25.6)	0.76 (0.60 to 0.85)

Data presented as mean ± SD, median (25–75), or stated otherwise.

CV: coefficient of variation; 95%CI: confidential interval; ICC: intraclass correlation coefficient for average measures; 6MWT: 6-min walk test; HR: heart ration; RR: respiratory rates; SBP: systolic blood pressure; DBP: diastolic blood pressure.

^†^Statistical difference (p < 0.05) from First 6MWT (paired T-test for parametric data or related-samples Wilcoxon Rank Test for non-parametric data).

^††^Significant desaturation after the test was considered when SpO_2_ decreased ≥ 5%.

**Figure 1:**
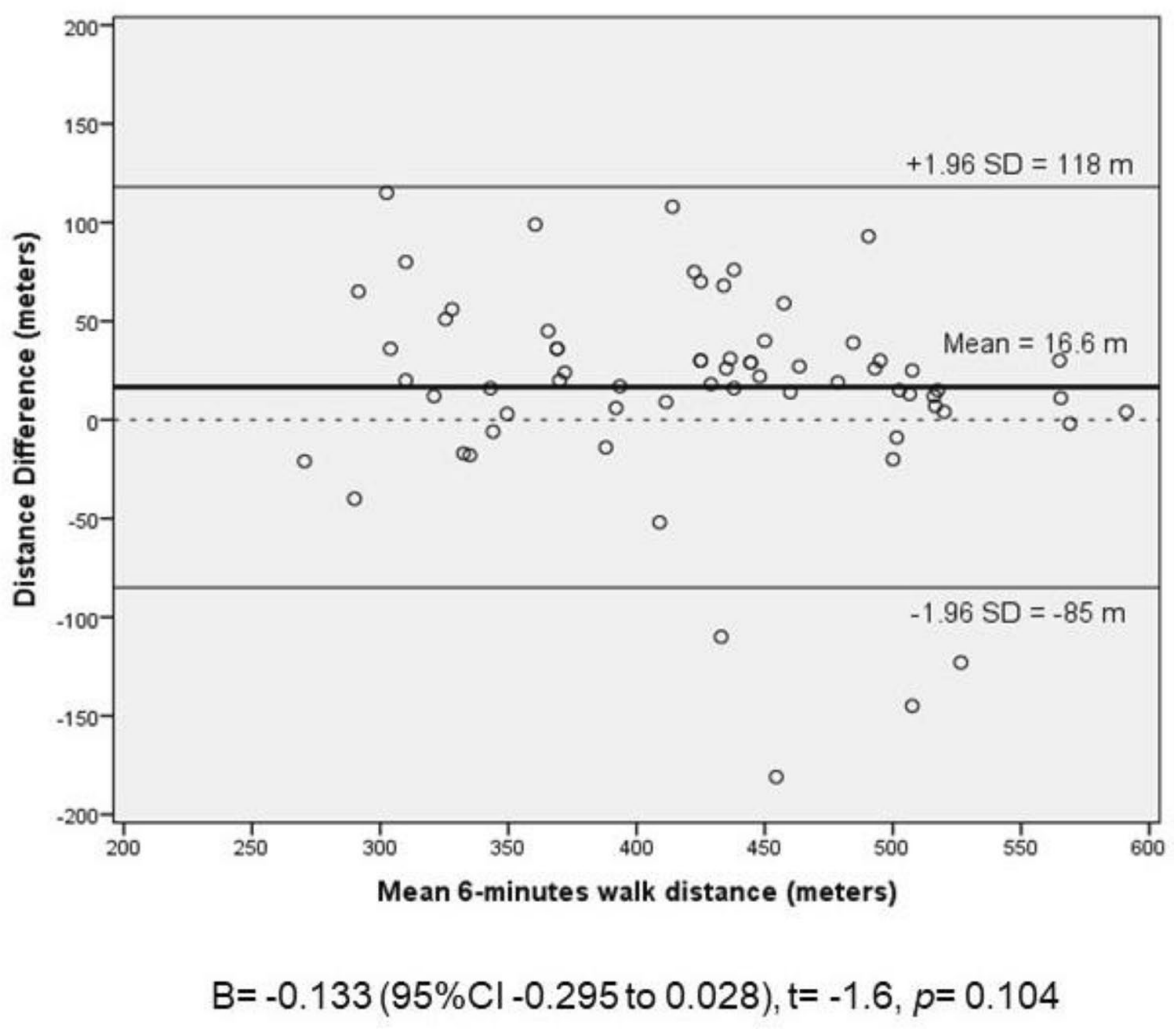
Bland–Altman plot of agreement in the distance walked between the 6-min walk tests (6MWTs) in subjects with non-cystic fibrosis bronchiectasis. Linear regression analyses of the distance difference and mean 6-min walked distance.

Figure 1 presents the Bland–Altman plot showing that there was an agreement in the distance walked between the two 6MWTs. The mean difference of walked distances was 16.6 m, presenting a limit of agreement between − 85 and 118 m. The regression analysis in Bland–Altman analyses was not significant [B = − 0.133 (95%CI − 0.295 to 0.028), t = − 1.6, *p* = 0.104)], confirming the agreement between test repeatability.

Table 3 shows that dyspnea and leg fatigue presented a moderate level of agreement between 6MWTs.

**Table 3:** Borg scores reproducibility.

	First 6MWT	Second 6MWT	Kappa^a^
At-rest Borg dyspnea score	0 (0–0)	0 (0–0)	0.61
Post-test Borg dyspnea score	2 (0.5–3)	2 (0.5–3)	0.62
At-rest Borg leg-fatigue score	0 (0–0)	0 (0–0)	0.66
Post-test Borg leg-fatigue score	0 (0–3)	0.5 (0–2.25)	0.78

Data presented as median (25–75).

6MWT: 6-min walk test.

^a^Weighted kappa coefficient of agreement.

**Figure 2:**
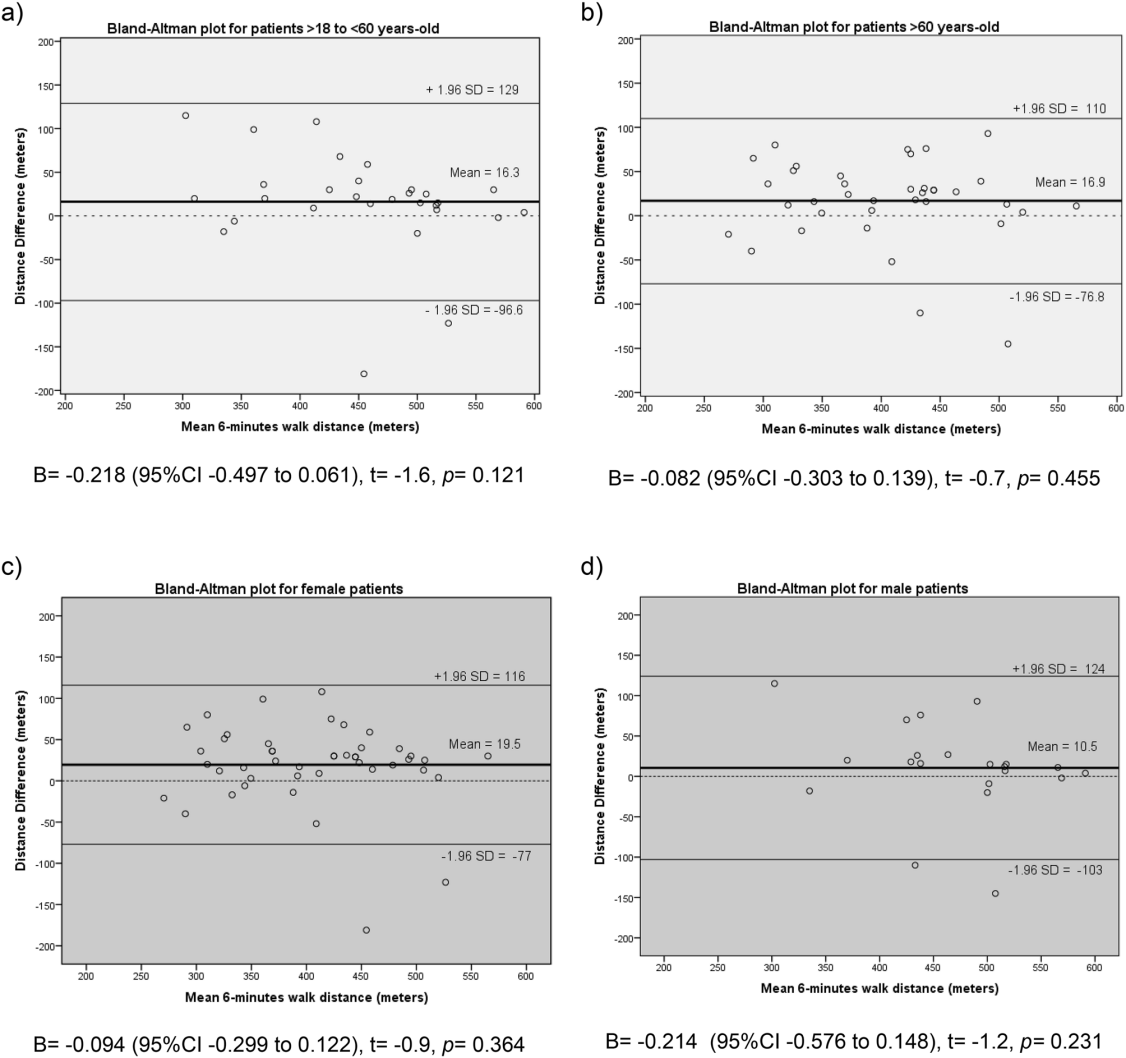
Bland–Altman plot of agreement in the distance walked between the 6-min walk tests (6MWTs)—groups subclassified by age (**a**) under and (**b**) above 60 years old; groups subclassified by sex (**c**) female and (**d**) male. Linear regression analyses of the distance difference and mean 6-min walked distance.

Significant association was observed between mean walked distance and BSI, indicating that the more severe the clinical condition, the shorter the walked distance (r = − 0.320, *p* = 0.009). In the sub-group analyses presented in Table 4, the distribution of disease severity was similar in both age and sex subclassification. Besides mean walked distance correlated negatively with age (r = − 0.461, *p* < 0.001), meaning that younger individuals walked a greater distance, the CVs were similar between tests in both young and old groups (Table 4). We also investigated the effect of sex and, although both groups presented similar CV, only the women’s group presented a reduced walked distance in the second test when compared to the first test (Table 4). Bland–Altman plot demonstrated that there is an agreement in the walked distances between the two 6MWTs in the age and sex subclassification analyses due to a non-significant linear regression, besides large limits of agreements (Fig. 2).

**Table 4:** 6-min-walk test results and reproducibility relative to age and sex differences of the participants.

Variables	Subclassification by age		Subclassification by sex	
	> 18 to < 60 years old	> 60 years old	Female	Male
n (%)	29 (44%)	37 (56%)	45 (68%)	21 (32%)
Age, years	39 ± 15	67 ± 5	55 ± 18	53 ± 16
Sex, men/women	10/19	11/26	–	–
Body Mass Index, kg/m^2^	25.4 ± 5.7	26.1 ± 5.0	26.0 ± 5.6	25.2 ± 4.5
FEV_1_, L	1.21 (0.88–2.00)	1.14 (0.89–1.45)	1.04 (0.81–1.24)	1.56 (1.30–2.07)*
FEV_1_, % predicted	43.3 ± 14.4	48.2 ± 13.3	47.0 ± 14.3	44.1 ± 13.0
FVC, L	2.30 ± 1.05	1.92 ± 0.66	1.82 ± 0.69	2.66 ± 0.95*
FVC, % predict	58.6 ± 15.3	62.0 ± 14.6	61.8 ± 15.8	57.7 ± 12.5
FEV_1_ post-bronchodilator, liters	1.33 (0.97–1.81)	1.22(0.93–1.47)	1.14 (0.86–1.34)	1.55 (1.31–1.79)*
FVC post-bronchodilator, liters	2.24 ± 0.82	1.91 ± 0.56	1.80 ± 0.53	2.60 ± 0.71*
BSI low/moderate/high score, n	11/5/13	7/14/16	11/10/24	7/9/5
**6MWT distance, m**				
First 6MWT	461 ± 74	413 ± 74*	415 ± 73	474 ± 71*
Second 6MWT	444 ± 90	396 ± 80*	395 ± 80	463 ± 86*
CV (95%CI), %	10.1 (5.2 to 13.5)	8.7 (6.6 to 10.5)	9.2 (6.4–11.2)	9.8 (4.2–13.2)
Paired *p*-value	0.139	0.038	0.011	0.416
**Post-6MWT HR, beats/min**				
First 6MWT	108 ± 15	115 ± 20*	113 ± 16	109 ± 23
Second 6MWT	105 ± 19	118 ± 18*	113 ± 17	111 ± 23
CV (95%CI), %	12.6 (7.1–18.0)	7.8 (3.7–11.8)	9.9 (5.5–14.3)	10.7 (5.4–16.0)
Paired *p*-value	0.678	0.120	0.867	0.544
**Post-6MWT RR, breaths/min**				
First 6MWT	23 ± 4	26 ± 5*	25 ± 5	24 ± 5
Second 6MWT	23 ± 4	28 ± 5*	26 ± 5	25 ± 6
CV (95%CI), %	5.4 (2.4–7.2)	9.8 (7.1–12.0)	7.7 (5.0–9.6)	9.2 (5.2–11.9)
Paired *p*-value	0.699	0.043	0.145	0.410
**Oxygen desaturation, %**^a^				
First 6MWT	0 (− 2 to 1)	− 1 (− 4 to -1)	− 1 (− 3 to 0)	− 1 (− 3 to 0)
Second 6MWT	0 (− 1 to 1)	− 1 (− 5 to 0)*	0 (− 2 to 0)	− 1 (− 5 to 0)

Data presents as mean ± SD, median (25–75) or stated otherwise.

Statistical analyses: Paired t-Test for intra-group comparison between first and second 6MWT. *Statistical difference (p < 0.05) between subclassifications: > 18 to < 60 versus > 60 years-old and female versus male; non-paired t-Test for parametric data and Mann–Whitney U test for non-parametric data.

CV: coefficient of variation; 95%CI: confidential interval; RR: respiratory rate; HR: heart rate; 6MWT: 6-min walk test; FEV_1_: forced expiratory volume; FVC: forced vital capacity; BSI: Bronchiectasis Severity Index.

^a^SpO_2_ difference between before and after the 6MWT.

## Discussion

The present study shows that the distance walked in two consecutive 6MWTs with a rest interval of 1 h between tests was reproducible in NCFB subjects. However, the distance walked in the second test was reduced when compared to the first test, which seems to be associated to clinical signs and disease severity indicating the need of a longer rest interval before repetition. In addition, there was a decent agreement in Borg dyspnea and leg fatigue response between tests.

The high level of agreement (ICC 0.88) indicates that there is a concordance between the two 6MWTs in the studied population. Bland–Altman plot shows that there are not many values below or above the mean difference distance of each subject evaluated. However, it presented a large limit of agreement from − 85 to 118 m, which shows that 95% of individuals with NCFB could vary from − 85 to + 118 m from the mean distance. Repeatability of 6MWT was also investigated in adults with NCFB by Lee et al.^13^ and it was observed an ICC of 0.95 with a shorter limit of agreement (− 35 to 75). These shorter limits could be related to the narrow range of age in Lee’s study participants (58 to 80 years old) when compared to the wide range in ours (18 to 81 years old). Ziegler et al.^14^ investigating the repeatability of 6MWT in cystic fibrosis adult patients observed an agreement between two consecutive tests (ICC = 0.94) with a large limit of agreement (− 75 to 62 m). Hernandes et al.^15^ observed that 6MWT in Chronic Obstructive Pulmonary Disease (COPD) patients was reproducible (ICC = 0.93) but also presented a large limit of agreement (− 67 to 120 m). Repeatability was also observed in two different Step tests in adult and old bronchiectasis patients (Chester Step Test and Modified Incremental Step Test), with a rest interval of 30 min^16^. In an overall analyses, the 6MWT presented a consider repeatability in NCFB.

In the present study, 79% of the subjects walked less in the second test from − 3 to − 115 m. Of these, 25% walked 50 to 115 m less and 40% walked 20 to 49 m less in the second 6MWT. On the other hand, just 6 subjects walked over 25 m in the second test. In a recent investigation with NCFB adults, Lee et al.^13^ observed that the subjects who walked a mean of 20 m further in the second 6MWT, with a rest period of 30 min, represented a small learning effect. The higher distance observed in Lee’s study could be related to a better clinical condition of the patients (mild to moderate lung function impairment and mean walked distance of 551 ± 90 m) when compared to our study. Considering the longer distance performed in the test, our participants walked 444 ± 77 m. This shorter distance walked when compared to Lee’s findings, could be due to a more severe clinical condition of the patients commonly followed in our Pneumology Ambulatory Care, as observed by BSI classification, as well as associated to other socio-environmental factors^17,18^. In another referral hospital in Brazil, using Step Test protocol, Camargo et al.^16^ showed that there were no differences regarding number of steps, time to perform, and vital signs between first and second assessments in NCFB subjects. In relation to walked distances, based on the minimal important difference (MID) of 25 m in patients with chronic respiratory disease^5,19^, our study observed that a very small group of subjects walked over of this threshold in the second test, which could be considered as a significant learning factor. The performance of 2 practice walks has been advocated by some centers because of the training effect related to factors such as anxiety and coordination^5,9^.

Furthermore, the shorter distance walked in the second test could be related to an insufficient rest interval for this specific population, even respecting the recovery period suggested by ATS guidelines^5,9^. In the present study, clinical signs as blood pressure and respiratory rate observed before starting the second test were significantly higher, suggesting that the participants were not fully recovered. Also, heart rate at the end of the first test was significantly higher in the group of patients who walked a reduced distance when compared to the group that walked further (115 ± 17 versus 99 ± 17 bpm, respectively; data not shown). Also, we observed that most of the patients (73%) presented a moderate to severe clinical condition based on BSI. The severity of the disease was associated to reduced walk distance, mainly in the second test (r = − 0.330, p < 0.007). Previous data of our group showed that young NCFB patients presented reduced 6-min walk distance when compared to older patients, which indicates that the more severe the disease the earlier the symptoms^7^. The reduced walk distance observed in the second test could be a characteristic of the NCFB patients besides available studies supporting a learning effect across all chronic respiratory disease based on COPD studies^20^. It suggests that NCFB patients may need a longer time to rest according to clinical signs and disease severity.

Regarding the age of the participants which range from 18 to 81 years old, it was observed a significant inverse correlation with walked distance. As expected, subjects above 60 years old walked significantly less when compared to younger subjects. Corroborating our data, NCFB patients aging above 50 years old walked significantly less when compared to younger subjects^21^. In the present study, shorter distances walked in the second test were observed not just in the old subjects, but also in the young subjects. In previous study of our group, younger NCFB patients (mean of 41 years old) were mostly in 6-min walked distance predicted lower limit compared to older patients (mean of 61 years old)^7^. These data reinforce the conclusion that one-hour of rest interval could be not enough to allow a patient with NCFB to return to their basal state, which could be due to pathophysiology condition of the disease.

Moreover, sex influenced the distance walked in the test repetition. Besides the expected difference in lung function and walked distance between women and men, women presented a significant reduction in walked distance in the second test when compared to the first test (mean reduction of 19.5 m), a difference not observed in men’s group (mean reduction of 10.5 m). In a study with Chinese subjects with bronchiectasis, women walked significantly less than men^21^. Besides the prevalence of bronchiectasis is higher in males, the disease is more severe in females due to the differences in physical and biological mechanisms, such as smaller lungs, earlier bacterial colonization, nutritional deficiencies, estrogen/progesterone modulate cilia beat affecting the mucociliary escalator and delayed diagnosis^22^.

Our study has some limitations. The participants presented a wide range of age and a small sample size which may contribute to the large limit of agreement observed. Future studies with a large samples size and a narrow age range is necessary to redefine the response to 6MWT in NCFB and to better generalize the test outcomes to this group of chronic lung disease.

In conclusion, our data could indicate repeatability of two 6MWT in adults with NCFB. However, vital signs should be carefully monitored and used to determine if the patient is fully recovered to perform the second 6MWT, besides the rest interval of 1 h between test, as well as the disease severity classified by BSI. The use of 6MWT to investigate exercise capacity is a secure, fast, and low-cost strategy to screening the NCFB subjects, providing for the health care team an important prognostic information for patient management.

## References

[1] McShane PJ, Naureckas ET, Tino G, Strek ME (2013). Non-cystic fibrosis bronchiectasis. Am. J. Respir. Crit. Care Med..

[2] Rosen MJ (2006). Chronic cough due to bronchiectasis: ACCP evidence-based clinical practice guidelines. Chest.

[3] Bar-Yoseph R (2019). Exercise capacity in patients with cystic fibrosis vs. non-cystic fibrosis bronchiectasis. PLoS ONE.

[4] Kadikar A, Maurer J, Kesten S (1997). The six-minute walk test: A guide to assessment for lung transplantation. J. Heart Lung Transplant..

[5] Holland AE (2014). An official European respiratory society/American thoracic society technical standard: Field walking tests in chronic respiratory disease. Eur. Respir. J..

[6] Solway S, Brooks D, Lacasse Y, Thomas S (2001). A qualitative systematic overview of the measurement properties of functional walk tests used in the cardiorespiratory domain. Chest.

[7] Jacques PS, Gazzana MB, Palombini DV, Menna Barreto SS, Dalcin PDT (2012). Six-minute walk distance is not related to quality of life in patients with non-cystic fibrosis bronchiectasis. J. Bras. Pneumol..

[8] Chalmers JD (2014). The bronchiectasis severity index an international derivation and validation study. Am. J. Respir. Crit. Care Med..

[9] Crapo RO (2002). ATS statement: Guidelines for the six-minute walk test. Am. J. Respir. Crit. Care Med..

[10] Jay SJ, Enright P (2000). Reference equations for the six-minute walk in healthy adults [1] (multiple letters). Am. J. Respir. Crit. Care Med..

[11] Pereira CA (2002). Espirometria. J. Pneumolo.

[12] Bland, M. How should I calculate a within-subject coefficient of variation? https://www-users.york.ac.uk/~mb55/meas/cv.htm (2006).

[13] Lee AL (2015). Field walking tests are reliable and responsive to exercise training in people with non’cystic fibrosis bronchiectasis. J. Cardiopulm. Rehabil. Prev..

[14] Ziegler B, Rovedder PME, Oliveira CL, e Silva FDA, Dalcin PDTR (2010). Repeatability of the 6-minute walk test in adolescents and adults with cystic fibrosis. Respir. Care.

[15] Hernandes NA (2011). Reproducibility of 6-minute walking test in patients with COPD. Eur. Respir. J..

[16] Camargo AA, Lanza FC, Tupinambá T, Corso SD (2013). Reproducibility of step tests in patients with bronchiectasis. Braz. J. Phys. Ther..

[17] Taylor-Robinson DC (2014). Low socioeconomic status is associated with worse lung function in the Danish cystic fibrosis population. Eur. Respir. J..

[18] Quanjer PH (2015). Low socioeconomic status and lung function. Eur. Respir. J..

[19] Lee AL (2014). Minimal important difference in field walking tests in non-cystic fibrosis bronchiectasis following exercise training. Respir. Med..

[20] Holland AE, Spruit MA, Singh SJ (2015). How to carry out a field walking test in chronic respiratory disease. Breathe.

[21] Guan WJ (2015). Six-minute walk test in Chinese adults with clinically stable bronchiectasis: Association with clinical indices and determinants. Curr. Med. Res. Opin..

[22] Vidaillac C, Yong V, Jaggi T, Soh M, Chotirmall S (2018). Gender differences in bronchiectasis: A real issue?. Breathe.

